# Genetic association of glutathione peroxidase-1 with coronary artery calcification in type 2 diabetes: a case control study with multi-slice computed tomography

**DOI:** 10.1186/1475-2840-6-23

**Published:** 2007-09-07

**Authors:** Masami Nemoto, Rimei Nishimura, Takashi Sasaki, Yoshito Hiki, Yumi Miyashita, Makiko Nishioka, Kei Fujimoto, Toru Sakuma, Toya Ohashi, Kunihiko Fukuda, Yoshikatsu Eto, Naoko Tajima

**Affiliations:** 1 Division of Diabetes, Metabolism and Endocrinology, Department of Internal Medicine, Jikei University School of Medicine, Tokyo, Japan; 2 Department of Gene Therapy, Institute of DNA Medicine, Jikei University School of Medicine, Tokyo, Japan; 3 Department of Radiology, Jikei University School of Medicine, Tokyo, Japan

## Abstract

**Background:**

Although oxidative stress by accumulation of reactive oxygen species (ROS) in diabetes has become evident, it remains unclear what genes, involved in redox balance, would determine susceptibility for development of atherosclerosis in diabetes. This study evaluated the effect of genetic polymorphism of enzymes producing or responsible for reducing ROS on coronary artery calcification in type 2 diabetes (T2D).

**Methods:**

An index for coronary-arteriosclerosis, coronary artery calcium score (CACS) was evaluated in 91 T2D patients using a multi-slice computed tomography. Patients were genotyped for ROS-scavenging enzymes, *Glutathione peroxidase-1 (GPx-1)*, *Catalase, Mn-SOD*, *Cu/Zn-SOD*, as well as SNPs of *NADPH oxidase *as ROS-promoting elements, genes related to onset of T2D (*CAPN10, ADRB3, PPAR gamma, FATP4*). Age, blood pressure, BMI, HbA_1c_, lipid and duration of diabetes were evaluated for a multivariate regression analysis.

**Results:**

CACS with Pro/Leu genotype of the *GPx-1 *gene was significantly higher than in those with Pro/Pro (744 ± 1,291 vs. 245 ± 399, respectively, *p *= 0.006). In addition, genotype frequency of Pro/Leu in those with CACS ≥ 1000 was significantly higher than in those with CACS < 1000 (45.5% vs. 18.8%; *OR *= 3.61, *CI *= 0.97–13.42; *p *= 0.045) when tested for deviation from Hardy-Weinberg's equilibrium. Multivariate regression analyses revealed that CACS significantly correlated with *GPx-1 *genotypes and age.

**Conclusion:**

The presence of Pro197Leu substitution of the *GPx-1 *gene may play a crucial role in determining genetic susceptibility to coronary-arteriosclerosis in T2D. The mechanism may be associated with a decreased ability to scavenge ROS with the variant *GPx-1*.

## Background

Evidence from large-scale clinical trials such as the Multiple Risk Factor Trial (MRFIT) [1] and the Hisayama study [2] demonstrates that mortality from coronary artery disease in patients with type 2 diabetes (T2D) is three to five times higher than in individuals with normal glucose tolerance. Therefore, existence of coronary artery disease should be considered as an adverse prognostic factor for patients with T2D. Thus, elucidation of the mechanism by which atherosclerosis occurs and develops in patients with T2D will lead to improving the prognosis of T2D patients.

Atherosclerosis is a multi-factorial disorder. Even if T2D patients are exposed to hyperglycemia to the same degree, two types of individuals can be observed; one in whom arterial sclerosis develops strongly and the second group in whom arterial sclerosis remains minor. Genetic susceptibility is thought to be the factor determining the onset of disease in those affected by hyperglycemia, especially the condition in which strong accelerators such as reactive oxygen species (ROS) accumulate. Tissue damage mediated by ROS, or oxidative stress, is implicated as a potential molecular mechanism leading to the development of atherosclerosis [3]. It is particularly important to maintain the redox milieu by balancing production and degradation of ROS, the so-called redox balance. This balance can be altered by either a reduction in the ability to degrade ROS but also by an increase in ROS production, resulting in excess ROS accumulation, inducing a redox imbalance, ultimately leading to tissue injury.

There are many studies focusing on the mechanism responsible for ROS overproduction but few of these have investigated the whole mechanism of redox balance. It is important to answer the question whether the degree of arterial sclerosis progresses strongly in diabetic subjects with genetic characteristics associated with low ROS degradation. Therefore, we thought it would be helpful for identification of the potentially susceptible genes to examine SNPs of genes involved in ROS metabolism in patients with T2D.

In this study, we examined T2D patients and evaluated their degree of coronary artery calcification by coronary artery calcium score (CACS) using multi-slice computed tomography (MSCT), which could provide highly quantitative evaluation of the degree of progression of arteriosclerosis. We investigated the relationship between CACS, and the redox-related genes and the genes associated with the onset of T2D to clarify the role of these genes in the mechanisms responsible for the onset of atherosclerosis in T2D.

## Methods

### Patients and clinical evaluation

Ninety-one patients with T2D (65 men and 26 women; mean age, 59 years; mean disease duration, 11.9 years) were enrolled in this study from November 2003 to December 2005. Patients were admitted to the Hospital of the Division of Diabetes Metabolism and Endocrinology at Jikei University School of Medicine, Tokyo, Japan.

Patients with T2D were diagnosed in accordance with the Japan Diabetes Society criteria, and were treated by either diet alone, oral agents or insulin. The study protocol was approved by the Ethics Committee of the Jikei University School of Medicine. Written informed consent was obtained from all patients before their participation in the study.

Medical history including family history, smoking status and duration of diabetes were recorded. Gender, body mass index (BMI) and blood pressure were also recorded. Fasting blood was examined for total cholesterol, triglyceride, HDL-cholesterol, plasma glucose, and HbA_1c _values.

### Determination of coronary artery calcification

The intensity and the extent of coronary artery calcification was determined by 16-slice MSCT (SOMATOM Sensation 16; Siemens Medical Solutions, Germany). Cardiac image data for coronary scan was acquired with retrospective ECG gating. The range of the entire heart was covered within a single breath hold (15–20 seconds). The rotation speed of the gantry was set as 0.42 sec/rotation and the helical pitch was set as 2.8. For image reconstruction, the slice thickness was set as 3.0 mm for quantification of CACS. CACS was calculated according to the algorithm suggested by Agaston et al as follows [4][5]. First, an area of interest was set in the calcification area of the coronary artery in each slice, and the calcification was considered significant when the CT value was 130 Hounsfield Units or over and the calcification area was 0.51 mm^2 ^(two pixels) or over. The highest CT value in each area, including significant calcification was weighted as 130–199HU = 1, 200–299HU = 2, 300–399HU = 3 and 400HU or over = 4. The calcification score in the area was obtained by multiplying the calcification area by the assigned weight, and the total calcification score in each area was the calcification score for the patient.

### Genotyping

In this study, we analyzed a total of 9 genes for the following two categories (Table 1): (a) genes to be associated with the onset of T2D and/or insulin resistance; and (b) genes for enzymes involved in the production and degradation of ROS. Genomic DNA was extracted from peripheral blood using FlexiGene (Qiagen, Hilden, Germany) following the manufacture's protocol. All single nucleotide polymorphisms (SNPs) were determined using PCR-RFLPs method: the Pro197Leu variant ascribed to the cSNP in exon 2 of the *Glutathione peroxidase-1 *(*GPx-1*) gene (rs1050450) [6], the A/T variation at position -89 in the promoter region of the *Catalase *gene [7], the Ala16Val variant in exon 2 of the *Mn-SOD *gene (rs4880) [8], the A/C substitution at position -34 in intron 3 of the *Cu/Zn-SOD *gene [9], the C/G variation at position +242 in exon 4 of the *NADPH oxidase *gene; *Cytochrome b light chain *(*CYBA*) gene (rs4673) [10], the A/G intronic iSNP of the *Calpain 10 *(*CAPN10*) gene (rs3792267) [11], the Try64Arg variant in exon 1 of the *β3-adrenergic receptor *(*ADRB3*) (rs4994) [12], the Pro12Ala variant in exon 1 of the *Peroxisome proliferator activated receptor γ *(*PPARγ*) (rs1801282)[13] and the Gly209Ser variant in exon 3 of the *Fatty acid transport protein-4 *(*FATP4*) (rs2240953) [14]. The oligonucleotides and the restriction enzymes used for the PCR-RFLPs are shown in Table 1.

**Table 1:** List of sequences of PCR primers used

a.				
	Gene	Restriction Enzyme	Direction	Primers
	*CAPN10 *(A/G-iSNP43)	Nsp I	Sense	5' GCTGGCTGGTGACATCAGTG3'
			Antisense	5' TCAGGTTCCATCTTTCTGCCAG3'
	*ADRB3 *(Try64Arg)	Mva I	Sense	5' CGCCCAATACC-GCCAACAC3'
			Antisense	5' CCACCAGGAGTCCCATCACC3'
	*PPARγ *(Pro12Ala)	Bst UI	Sense	5' GCCAATTCAAGCCCAGTC3'
			Antisense	5'GATATGTTTGCAGACAGTGTATCAGTGAAGGAATCGCTTTCCG3'
	*FATP4 *(Gly209Ser)	Hpa II	Sense	5' GTGAGGTCCATGCCAGCCTG3'
			Antisense	5' CACCTGTGAAGCCCTTGGTCAG3'

b.				
	Gene	Restriction Enzyme	Direction	Primers

	*GPx-1 *(Pro197Leu)	Hae III	Sense	5' TTATGACCGACCCCAAGCTCA3'
			Antisense	5' ACAGCAGCACTGCAACTGCC3'
	*Catalase *(SNP-89)	Hinf I	Sense	5'AATCAGAAGGCAGTCCTCCC3'
			Antisense	5'TCGGGGAGCACAGAGTGTAC3'
	*Mn-SOD *(Ala16Val)	BsaW I	Sense	5' GCTGTGCTTTCTCGTCTTCAG3'
			Antisense	5'TGGTACTTCTCCTCGGTGACG 3'
	*Cu/Zn-SOD *(intron 3)	Hha I	Sense	5'CTATCCAGAAAACACGGTGGGCC3'
			Antisense	5'ATTGCCCAAGTCTCCAACATGC 3'
	*NADPH oxidase *(+242)	Rsa I	Sense	5'ACACTGAGGTAAGTGGGGGTGGCTCCTGT3'
			Antisense	5' TGCTTGTGGGTAAACCAAGGCCGGTG3'

Oligonucleotides used as PCR primers for the detection of each SNP. a) genes associated with the onset of type 2 diabetes and/or insulin resistance. b) genes related to the ROS-scavenging system. Enzymes represent restriction enzymes for detection of each RFLP of PCR product.

DNA amplification was carried out in a final volume of 20 *μ*L, using 0.1 *μ*g of genomic DNA and 1 nmol each of the primers. The four deoxynucleotides were included in a final concentration of 100 *μ*mol/L. The reaction buffer was prepared as recommended by the manufacturer. The amplification reaction was started by adding 0.5 units of *Taq *polymerase (TAKARA, Japan). Annealing, extension and denaturing were carried out using an automatic thermal cycler (DNA Engine^®^, Bio-RAD, Japan). The amplified products were digested at 37 or 60°C for 1 hour using 25 *μ*L of each PCR-amplified product with 10 units of the restriction enzyme (New England Biolabs, Inc., MA, USA and TAKARA, Japan). The digested products were then electrophoresed at 100V for one hour through a 3.5% agarose gel. The gels were stained with ethidium bromide and visualized by ultraviolet light to determine the SNPs.

### Analysis of genetic association

The CACS values were tested for differences among the patients with different genotypes of all candidate genes by the Student's t-test. Contribution of BMI, age, gender, duration, HbA_1c_, systolic blood pressure and LDL-cholesterol related to CACS was also evaluated by the multivariate regression analysis. The Student's t-test and the multivariate regression analyses were performed using Dr. SPSS II (SPSS, Chicago, IL, USA). *P *< 0.05 was considered to be statistically significant. Allele and genotype frequencies of cases (CACS ≥ 1,000) and controls (CACS < 1,000) were compared with values predicted by Hardy-Weinberg's equilibrium (HWE) using the χ^2 ^test. For this purpose, the frequency of the *GPx-1 *variant (Pro197Leu) allele and the genotype with Pro/Leu at the 197 position were compared between the controls and the cases. Calculation for the case-control study was performed using the DeFinetti computer program . All values in clinical tests were presented as mean ± SD.

## Results

Clinical characteristics of the patients are summarized in Table 2. In the genetic association study, our aim was to know whether the CACS would be different according to the genotypes of each gene. When compared among genotypes, the observed CACS showed no significant difference except at position 197 of the *GPx-1 *gene where proline is the major allele and leucine is the minor allele, as shown in Table 3. Heterozygotes with Pro197Leu of the *GPx-1 *gene had significantly higher CACS than homozygotes of proline allele (744 ± 1,291 vs. 245 ± 399, respectively, *p *= 0.006). This result shows a possibility that the Pro197Leu variation of the *GPx-1 *gene is genetically associated with an increase in CACS, or acts as a marker for coronary sclerosis. Significant differences in CACS of Pro/Leu at position 197 of GPx-1 also suggest that the genetic mode is dominant. There was no significant difference in the clinical characteristics between patients with Pro/Pro and Pro/Leu (Table 4). Confounding factors were further examined for association with CACS by multiple regression analysis where the dependent variable was CACS, and the explanation variables were clinical parameters and candidate genes. The results of the analysis also showed a significant correlation between CACS and two variables: *GPx-1 *genotype (standardization coefficient = 0.340, *p *= 0.004) and age (standardization coefficient = 0.257, *p *= 0.042).

**Table 2:** Clinical characteristics of patients with type 2 diabetes.

Age (years)	60 ± 8 (range, 42 – 81)
Duration of diabetes (years) BMI (kg/m^2^)	11.6 ± 8.9 (range, 0 – 42) 24.4 ± 3.7
LDL-cholesterol (mmol/l)	3.37 ± 0.81
HDL-cholesterol (mmol/l)	1.31 ± 0.55
Triglyceride (g/l)	1.69 ± 1.15
Blood pressure (systolic) (mmHg)	137 ± 18
Blood pressure (diastolic) (mmHg)	80 ± 11
HbA_1c _(%)	8.2 ± 1.8
Smoking	40/91 (44.0%)

Data are mean ± standard deviation (SD) or frequencies (%).

**Table 3:** Results of association analyses between each genotype of candidate genes and coronary artery calcium score (CACS).

a.					
	Gene (SNPs)	Genotype	No. of patients	CACS (HU)	*p *value
	
	*CAPN10 *(A/G-iSNP43)	G/G	84 (92.3%)	362 ± 735	0.456
		G/A	7 (7.7%)	153 ± 150	
	*ADRB3 *(Try64Arg)	Try/Try	55 (61.8%)	377 ± 776	0.621
		Arg/Arg & Arg/Try	34 (38.2%)	301 ± 605	
	*PPARγ *(Pro12Ala)	Pro/Pro	85 (93.4%)	335 ± 686	0.580
		Ala/Pro & Ala/Ala	6 (6.6%)	502 ± 1049	
	*FATP4 *(Gly209Ser)	Gly/Gly	48 (52.7%)	346 ± 797	0.991
		Ser/Ser & Ser/Gly	43 (47.3%)	346 ± 605	
	
b.					

	Gene (SNPs)	Genotype	No. of patients	CACS (HU)	*p *value
	
	*GPx-1 *(Pro197Leu)	Pro/Pro	71 (78.0%)	245 ± 399	0.006 *
		Pro/Leu	20 (22.0%)	744 ± 1291	
	*Catalase *(SNP-89)	T/T	25 (27.5%)	319 ± 573	0.821
		A/T & A/A	66 (72.5%)	357 ± 758	
	*Mn-SOD *(Ala16Val)	Val/Val	49 (55.7%)	293 ± 669	0.440
		Val/Ala & Ala/Ala	39 (44.3%)	408 ± 756	
	*Cu/Zn-SOD *(intron 3)	C/C	34 (37.4%)	370 ± 788	0.805
		C/A & A/A	57 (62.6%)	332 ± 664	
	*NADPH oxidase *(exon 4)	C/C	79 (86.8%)	311 ± 724	0.224
		T/T & T/C	12 (13.2%)	579 ± 567	

Difference of CACS according to each genotype of candidates genes. a) genes that are associated with onset of type 2 diabetes. b) genes that are related to the redox balance. Student t-test; * : *p *< 0.05.

**Table 4:** Clinical characteristics of patients with Pro/Pro and Pro/Leu at GPx-1 gene.

	Pro/Pro	Pro/Leu	*p *value
Age(years)	59.4 ± 8.3	62.7 ± 8.9	0.136
Duration of diabetes(years)	11.3 ± 8.5	12.5 ± 8.8	0.609
BMI(kg/m^2^)	24.7 ± 3.9	24.2 ± 3.1	0.596
LDL-cholesterol(mmol/l)	3.39 ± 0.80	3.39 ± 0.83	0.978
HDL-cholesterol(mmol/l)	1.32 ± 0.65	1.31 ± 0.55	0.679
Triglyceride(g/l)	1.71 ± 1.16	1.63 ± 1.16	0.809
Blood pressure (systolic) (mmHg)	138 ± 19	132 ± 13	0.208
Blood pressure (diastolic) (mmHg)	81 ± 12	76 ± 8	0.119
HbA_1c _(%)	8.5 ± 1.8	7.8 ± 1.6	0.158
Smoking (%)	43.1	63.7	0.272

Data are means ± standard deviation (SD) or frequencies (%).

In order to confirm the genetic association between CACS and the *GPx-1 *gene, we next conducted a case-control study based on Hardy-Weinberg's law, comparing the genotype frequencies in accordance with the CACS value. When the patients were stratified by CACS as shown in Table 5a, a significant difference in distribution of the genotype frequency for the Pro197Leu cSNP at *GPx-1 *gene was observed. The frequency of the Pro/Leu heterozygote among the cases (CACS values ≥ 1,000) was significantly higher than that among the controls (CACS values < 1,000) (*OR *= 3.61; *CI *= 0.97 – 13.4; *p *= 0.045). Analysis for differences in the allelic distribution also revealed the same tendency (Table 5b, *p *= 0.06).

**Table 5:** Association between Pro197Leu of the *GPx-1 *gene and coronary artery calcium score (CACS)

a) Genotype distribution of *GPx-1*			
CACS	Pro/Pro	Pro/Leu	*p *value
0–999	65	15(18.8%)	0.045
1000 ≤	6	5(45.5%)	
			
b) Allele distribution of *GPx-1*			
CACS	Pro	Leu	*p *value

0–999	145	15(9.4%)	0.06
1000 ≤	17	5 (22.7%)	

Results of the case-control analysis for the allele and genotype frequencies of the *GPx-1 *gene. Genotype (a) and allele (b) frequencies were analyzed in the case-control study based on the HWE (De Finetti program). a) Analysis for the *GPx-1 *gene showed that the genotype frequency of Pro/Leu in those with CACS (≥ 1000) was significantly higher than that in those with CACS (0–999) and this association was confirmed by testing for deviation from Hardy-Weinberg's equilibrium (*OR *= 3.61; *CI *= 0.97–13.42; *p *= 0.045). b) The allele frequency of Leu in patients with CACS (≥ 1000) was also higher than that in those with CACS (0–999) and a trend for genetic association was appeared when testing for deviation from HWE (*OR *= 2.84; *CI *= 0.92–8.8; *p *= 0.06).

## Discussion

It has been established that patients with coronary artery calcification evaluated by MSCT are at high risk for coronary events [15]. As prevalence of coronary artery calcification and coronary events are significantly greater among patients with T2D than the non-diabetic controls [16], the mechanism and the traditional risk assessment for coronary atherosclerosis in patients with T2D must be refined.

Atherosclerosis appears to be a multifactorial disorder, in which the classical Framingham factors, aging and poorly controlled diabetes are independent risk factors for the development of atherosclerosis. However, it is still not easy to identify genetic factors for atherosclerosis among their confounding components even though knowledge of the human genome project has been established. In our attempt to identify genes associated with coronary artery sclerosis, we analyzed genes involved in the pathogenesis for development of T2D as well as genes of enzymes that are pertinent to determination of the redox balance. The sample size of this report was limited as a genetic association study because the X-ray examination was a prerequisite for participation. However, our present study is unique in that the predisposition for coronary artery sclerosis by altered function in redox-related genes was analyzed with using MSCT-assisted calcium scoring combined with known risk factors. MSCT-assisted evaluation of CACS represents now a standard index indicating intensity and extent of the coronary atherosclerosis in routine clinical practice. In addition to the calcifications, the atherosclerotic plaque and the obstructive lesion in coronary artery are major issues with the latest 64-MSCT. It would be interesting to investigate which observation including the properties of the plaque, calcifications and stenosis is strongly associated with the *GPx-1 *polymorphism as the information should provide further insights into the mechanism for development of coronary atherosclerosis.

There is growing evidence that excess generation of highly reactive free radicals, largely due to hyperglycemia, causes oxidative stress, which further exacerbates the development and progression of diabetes and its complications. Overproduction and/or insufficient removal of these free radicals result in vascular dysfunction, damage to cellular proteins, membrane lipids and nucleic acids [17]. GPx-1 represents the first identified mammalian selenoprotein [18], and our understanding in the metabolic regulation and function of this abundant selenoenzyme has greatly advanced during the past decade [19]. However, GPx-1 exerts a dual role in reactive nitrogen species (RNS)-related oxidative stress [20]. Intracellular and tissue levels of GPx-1 activity affect apoptotic signaling pathway, protein kinase phosphorylation, and oxidant-mediated activation of NFkappaB. Data are accumulating to link alteration or abnormality of GPx-1 expression to etiology of cardiovascular disease and diabetes [21]. The development of insulin resistance in mammals with elevated expression of an antioxidant enzyme and suggest that increased GPx-1 activity may interfere with insulin function by overquenching intracellular reactive oxygen species required for insulin sensitizing [22].

Results from our multivariate analysis and association study revealed the polymorphism of *GPx-1 *gene to be associated with MSCT-detected CACS on all the tested elements except aging. It has been reported that low activity in the GPx-1 of red blood cells is associated with an increased risk for the onset of cardiovascular events [23]. In addition, low activity in GPx-1 has also been implicated in promoting calcification of coronary arterial walls. More importantly, a recent paper investigating the common variant Pro197Leu of *GPx-1 *reported this variant to be associated with a 40% decrease in activity [24]. GPx-1 is expressed in endothelial cells and macrophages of normal and atherosclerotic vessels [25]. Therefore, individuals having the Pro197Leu variant at *GPx-1 *are assumed to have a genetically low level of *GPx-1 *activity at vascular wall and hence their vessels may be sensitive to oxidative stress. This may be attributable to the observed association between patients with the Pro197Leu variant and higher CACS in our present study.

Non-diabetic individuals were not examined in this study; but the multivariate analysis and the association study among T2D patients revealed a significant association between CACS and *GPx-1 *polymorphism. It has almost been established that production of ROS in patients with diabetes becomes excessive and results in oxidative stress. This may account for the observed association between CACS and the gene of the ROS-scavenging *GPx-1 *in T2D patients in spite of the lack of association with polymorphisms of the ROS-promoting enzymes. Further study could reveal a possibility of intervention to prevent atherosclerosis among T2D patients by adding sufficient activity of GPx-1.

## Conclusion

This study revealed a genetic association between the presence of the Pro197Leu variant of the *GPx-1 *gene with MSCT-detected coronary artery calcification. Functional relevance of a variant for the antioxidant enzyme may account for a critical role for changes in the redox balance in the pathogenesis of coronary atherosclerosis in T2D patients.

## Competing interests

The author(s) declare that they have no competing interests.

## Authors' contributions

MN, RN, TS, YH, YM, KF and NT carried out recruitment of patients. MN, YH, TO and YE carried out the molecular genetic studies. MN, TS and KF carried out Measurement of CACS by MSCT. MN, TS, RN, NT participated in the design of the study and performed the statistical analysis. All authors read and approved the final manuscript.
